# Ultra Compact and Broadband Fiber Facet Graphene Oxide Microlens Array Fabricated on a Beam‐Expanding Fiber Facet Using Femtosecond Laser Direct Writing Technique

**DOI:** 10.1002/nap2.70217

**Published:** 2026-07-29

**Authors:** Xiaoke Chen, Lin Ma, Linbin Bai, Tianqi Cheng, Zuyuan He

**Affiliations:** ^1^ State Key Laboratory of Photonics and Communications Shanghai Jiao Tong University Shanghai China; ^2^ Shanghai Optoweave Technology Co. Ltd. Shanghai China

**Keywords:** femtosecond laser direct writing, fiber facet device, graphene oxide, nanophotonics devices

## Abstract

We demonstrate an ultra‐compact fiber facet graphene oxide (GO) microlenses array fabricated through femtosecond laser direct writing (FLDW). Taking advantage of the broadband transparency, mechanical, and laser‐tunable optical properties of GO, the device realizes phase modulation in a 400 nm film, enabling multi‐focus beam splitting and focusing. A coreless fiber segment is fusion‐spliced to a standard single‐mode fiber to expand the mode field, and a high‐density array of 19 microlenses is integrated on the beam‐expanding fiber facet; the device achieved focal lengths ranging from 8.0 to 9.6 μm and numerical apertures (NA) from 0.60 to 0.74. Experimental results at 850, 1310, and 1550 nm demonstrate broadband compatibility. This single‐step fabricated, ultrathin device offers a compact scalable solution for fiber‐probe imaging, multi‐point sensing, micro‐optical manipulation and integrated nanophotonic systems.

## Graphical abstract

A 400 nm graphene oxide film on a beam‐expanding fiber facet is patterned by femtosecond laser direct writing into a 19‐lens microlens array. The device simultaneously splits a single input beam into multiple 0.67–0.74 NA focus spots with a broadband response from 850 to 1550 nm. This single‐step ultrathin platform offers a compact solution for multipoint sensing, fiber probe imaging, and integrated nanophotonics.

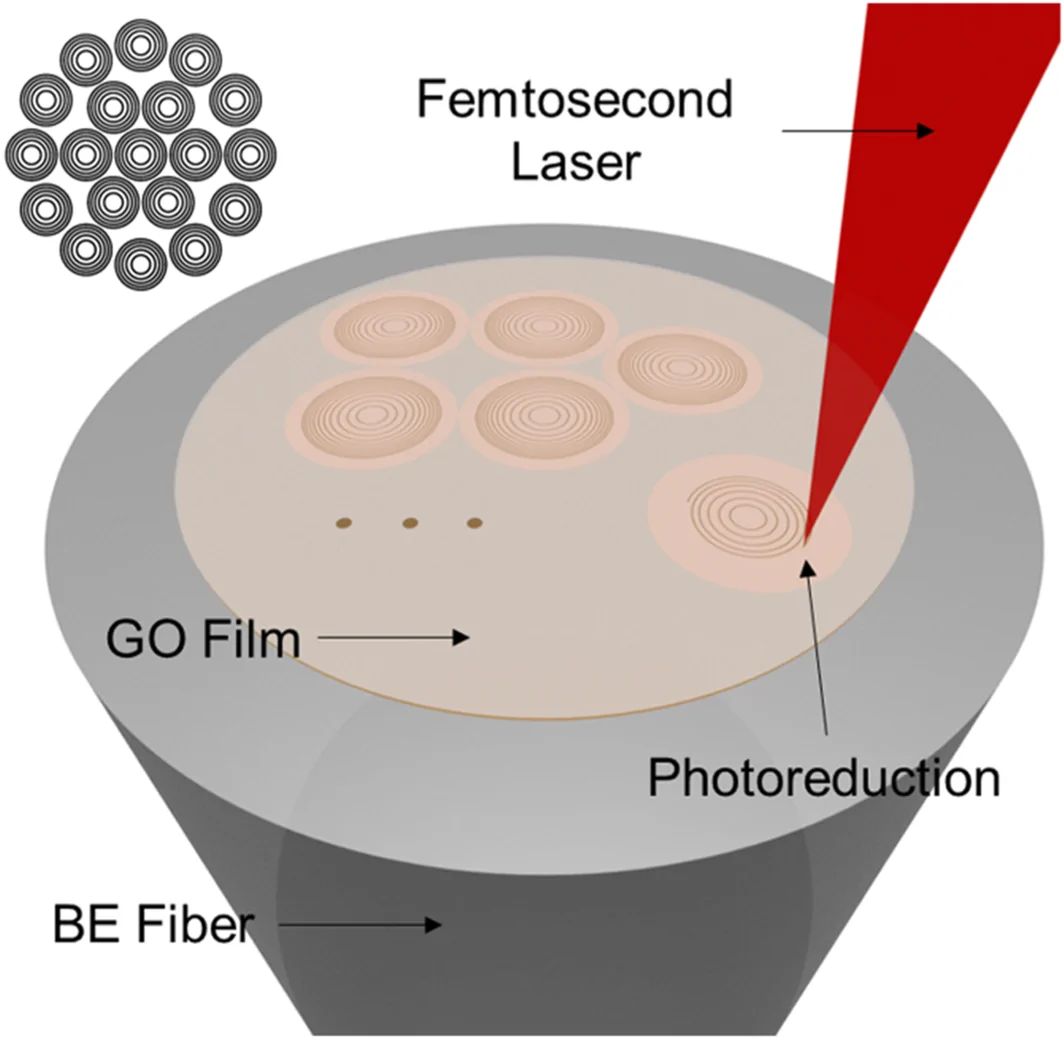

## 1 Introduction

As integrated photonics and miniaturized optical systems have been developed [1], devices that directly integrate photonics functionality on fiber facets have emerged as an important route toward compact optical control and miniaturization [2, 3]. Fiber‐facet devices preserve the intrinsic advantages of optical fibers, including low‐loss transmission, natural coaxial alignment, and packaging compatibility [4], while allowing on‐tip functionalities, such as focus [5], beam splitting [6], and shaping [7]. These capabilities make them attractive for fiber probes [8], parallel excitation [9], multi‐channel detection [10], optical sensing [11], and micro/nano manipulation [7]. Compared with free‐space micro‐optical assemblies, fiber‐facet‐integrated devices significantly reduce system complexity and improve operational stability, and therefore have became an active research focus in functionalized fiber and nanophotonic devices.

For beam splitting and multi‐focus generation, which are widely required in multi‐spot imaging [12] and micro/nano optical manipulation [7], existing approaches mainly rely on planar integrated photonic platforms and free‐space micro‐optics, including waveguide splitters [13], multimode interference (MMI) devices [14], directional couplers [15], diffractive optical elements [16], and micro optical element arrays [17]. Although these devices are well established in on‐chip photonic platforms, they generally rely on planar waveguide architectures and multi‐step lithographic fabrication, leading to restricted material platforms and limited design flexibility. Coupling them with fiber systems often requires additional alignment structures and packaging modules [18], increasing overall system complexity and limiting facet‐level integration. Free‐space microlens arrays can generate multiple focal spots, but usually involve bulky configurations and extended optical paths [19], which are not suitable for highly compact fiber‐based microsystems. Therefore, a fabrication strategy that enables the direct construction of compact fiber‐facet devices with combined focusing and beam‐splitting capability and simplified processing remains highly desirable.

Various fabrication routes have been explored for fiber–facet micro‐optics, including two‐photon polymerization 3D printing [20, 21], polymer microlens [22], metasurface nanolithography [23], and multilayer structuring [24]. Although these techniques support complex wavefront engineering, they commonly suffer from large structural thickness, complex processing flows, strong equipment dependence, or limited environmental robustness. Polymer‐based microlenses typically exhibit low refractive‐index contrast and thus require large feature heights to achieve sufficient phase modulation [25, 26], which restricts high‐numerical‐aperture operation and dense array integration. Fiber–facet metasurfaces provide precise phase control, but often rely on high‐cost nanofabrication techniques such as electron‐beam lithography or focused ion beam milling, facing challenges in fabrication efficiency, scalable area processing, and process stability. These limitations highlight the need for an ultrathin, high‐phase‐efficiency material platform and a direct‐write fabrication method suitable for fiber facets.

Graphene oxide (GO) and reduced graphene oxide (rGO) provide a promising platform for ultrathin diffractive optical devices [27, 28, 29, 30]. GO films exhibit broadband transparency [31, 32], mechanical flexibility [33], and chemical stability [34], and their optical constants can be continuously tuned through controlled reduction [35]. Under localized femtosecond laser irradiation, GO can be selectively converted to rGO [36, 37], producing pronounced refractive‐index and thickness contrast at submicrometer scale and enabling near π phase modulation within an ultrathin layer [38]. This high‐index‐contrast, ultrathin modulation mechanism removes the dependence on large structural thickness typical of polymer microstructures, offering intrinsic advantages for high‐NA, small‐footprint, and high‐density integrated diffractive optics [39, 40]. In addition, femtosecond laser direct writing is mask‐free, single‐step, and high‐resolution, and is well suited for in situ fabrication of functional microstructures on confined surfaces such as fiber facets.

Based on this material platform and fabrication approach, we propose and demonstrate a GO microlens‐array beam‐splitting device integrated on a fiber facet. A coreless fiber segment is fusion‐spliced to a single‐mode fiber to expand the mode field and provide a uniform enlarged output area. In our previous work, we demonstrated single‐lens GO devices on fiber facets with ultra‐high NA [41] and OAM beam generation [42]. Here, we extend this material platform to a multi‐element array configuration, addressing the additional challenges of beam expansion, array packing, and peripheral wavefront control. An ultrathin GO film is then transferred onto the facet, and concentric rGO ring structures are directly patterned by femtosecond laser writing to form diffractive microlens units. Dense array arrangement of these units enables facet‐level multi‐focus beam splitting and simultaneous focusing from a single input beam. The device is designed using diffraction theory to achieve multi‐spot focusing and energy distribution. A 19 high‐density GO microlens array is experimentally fabricated. Structural characterization confirms good uniformity of rGO thickness and phase modulation. Results show stable multi‐focus spots and well‐defined focusing behavior at 850 nm, as well as at two representative telecom wavelengths of 1310 and 1550 nm, indicating broadband compatibility.

Compared with polymer fiber microlenses and metasurfaces, the GO‐based facet platform enables ultrathin phase modulation using a single step femtosecond direct writing, reducing both device thickness and fabrication complexity. By combining mode‐field expansion with arrayed diffractive design, beam splitting and focusing are integrated on a single fiber facet, providing a compact and scalable solution for fiber‐probe imaging, multi‐point sensing, chip‐to‐fiber optical interfaces, and micro optical manipulation.

## 2 Theoretical Design

The designed fiber facet GO lens array is schematically shown in Figure 1a and consists of three parts. The first part is a standard single‐mode fiber (SMF, G652.D). The second part is a mode‐field expansion region, formed by fusion splicing a segment of coreless fiber with length l to the end of the SMF. The refractive index of the coreless fiber is equal to the SMF cladding's RI. The third part is the GO lens array, where a thin GO film is directly transferred onto the fiber facet, and a lens array is fabricated within the film using femtosecond laser direct writing. A representative 2 × 2 lens array is shown in the schematic.

**FIGURE 1:**
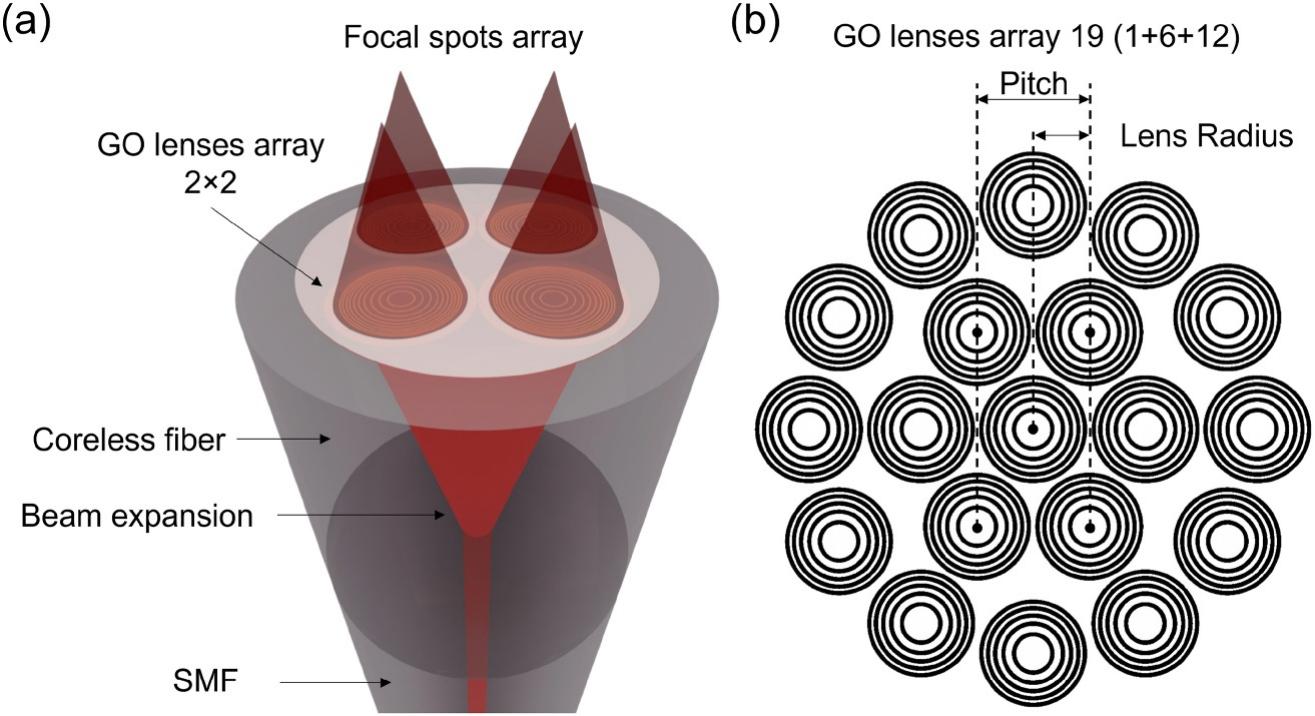
Schematic of fiber‐end GO microlens array: (a) structure: SMF, beam‐expanding coreless fiber, and microlenses with defined radius and pitch. Examples: 2 × 2 lenses array; (b) designed GO lenses array (1 + 6 + 12 layout), lens radius and lens pitch.

First, the mode‐field expansion section needs to be designed. The core diameter of the SMF is 9 μm, which provides a limited effective area on the fiber end face and restricts the integration of high‐density lens arrays. To overcome this limitation, a coreless fiber‐based mode‐field expansion scheme is adopted to enlarge the SMF output beam for lens array integration.

The mode‐field expansion is analyzed using the optical transfer matrix (ABCD matrix) method. The optical field emerging from the SMF end face is assumed to be a Gaussian beam with a mode field diameter (MFD) of $w_{0}$. The fusion‐splicing interface between the SMF and the coreless fiber is considered to be the beam waist. The propagation of the beam inside the coreless fiber follows the evolution of Gaussian beam. After propagating through a coreless fiber of length l, the wavefront can be described by a Gaussian beam model:

(1) $$E\left(r,z\right)=\frac{w_{0}}{w\left(z\right)}\exp\left(-\frac{r^{2}}{w\left(z\right)}\right)\exp\left(jkz+jk\frac{r^{2}}{2R\left(z\right)}-j\zeta \left(z\right)\right),$$

where w(z) is the beam radius, R(z) is the corresponding wavefront curvature radius and ζ(z) is the Gouy phase. At position *z*, the above parameters can be derived from the *q*‐parameter:

(2) $$q=\frac{Aq_{0}+B}{Cq_{0}+D}=\frac{q_{0}+ln_{1}/n_{2}}{n_{1}/n_{2}}=\frac{n_{2}}{n_{1}}q_{0}+l,\;\frac{1}{q\left(z\right)}=\frac{1}{R\left(z\right)}-j\frac{\lambda }{n\pi w^{2}\left(z\right)},$$

where $n_{1}$ and $n_{2}$ is the RI of core and clading of the SMF and ABCD is the propagation matrix of the interface between the SMF and the coreless fiber, as well as the transmission through the coreless fiber, $q_{0}=j\pi w_{0}^{2}/\lambda$ is the *q*‐parameter at beam waist.

Figure 2 presents both the calculated mode‐field expansion curves and the fabricated SMF–coreless fiber structure. The results indicate that the MFD increases monotonically with increasing coreless fiber length. However, in practical fabrication, the expansion is constrained by the 125 μm diameter of the ceramic ferrule. When the expanded MFD exceeds 125 μm, interference fringes appear near the beam edge due to reflections at the interface between the coreless fiber and the ceramic ferrule, which degrades the quality of the effective beam. To achieve a sufficiently large mode‐field radius while avoiding edge interference effects, a coreless fiber length of 1000 μm is selected. Under this condition, the MFD expands from 6.7 μm to approximately 107.5 μm.

**FIGURE 2:**
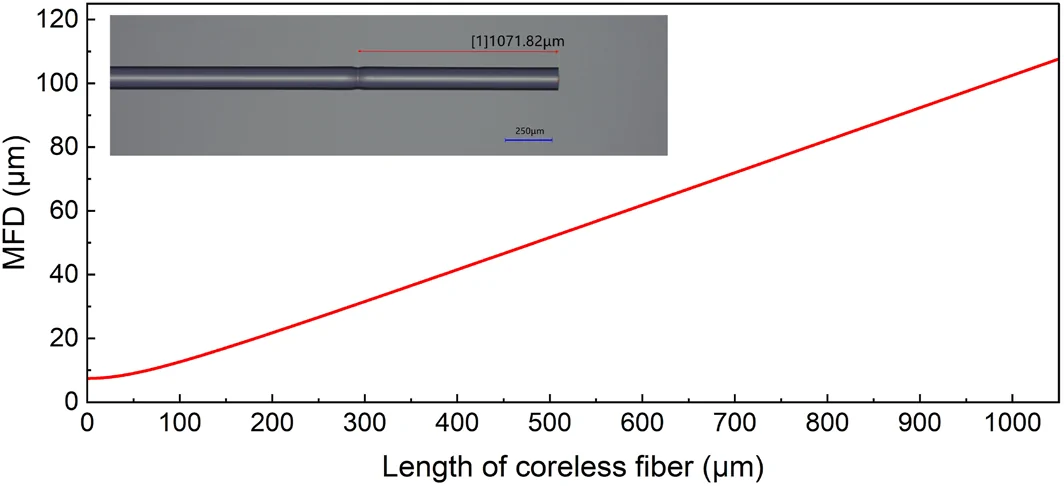
Expansion of MFD with coreless fiber length.

The second part of the design focuses on the GO lens array. According to the focusing principle of GO lenses, constructive interference along the optical axis is required to achieve on‐axis focusing. Because of the outstanding differences in RI between GO and rGO, concentric rGO ring structures can simultaneously modulate both the amplitude and phase of the incident optical field.

The rGO ring zones of a single GO lens are optimized based on the Rayleigh–Sommerfeld (R–S) diffraction theory to achieve efficient focusing. Considering a given incident field $U_{in}$, to design a GO lens with focal length f, the optical intensity at focal spot can be expressed as follows:

(3) $$I={\left|\frac{1}{2\pi }\iint_{\mathrm{lens}}U_{in}\left(ik-\frac{1}{\delta }\right)\frac{\exp\left(ik\delta \right)}{\delta^{2}}ds\right|}^{2},$$

where r is the radial coordinate on diffractive plane and $\delta =\sqrt{r^{2}+f^{2}}$ is the distance from the diffractive plane to the focal point. The critical positions of the rGO rings *r*_*i*_ are defined by the condition where the intensity gradient is zero:

(4) $$r_{i}\left|\frac{dI}{dr}\right.{|}_{r=r_{i}}=0,$$

indicating radial critical constructive positions of the rings to achieve interference at focal spot.

Based on this design principle, a GO lens array operating at a wavelength of 850 nm is fabricated in this work. The array consists of 19 individual lenses arranged in a 1 + 6 + 12 configuration, including a central lens, a middle ring of six lenses arranged in a hexagonal close‐packed structure, and an outer ring of 12 lenses arranged in a dodecagonal pattern, as shown in Figure 1b. The center‐to‐center pitch between adjacent lenses is twice the designed lens radius. To demonstrate the feasibility of high‐density integration on the fiber facet, each lens is designed with a radius of 20 μm and a focal length of 9 μm.

The lens performance was simulated by R–S diffraction theory, and the results are shown in Figure 3.

**FIGURE 3:**
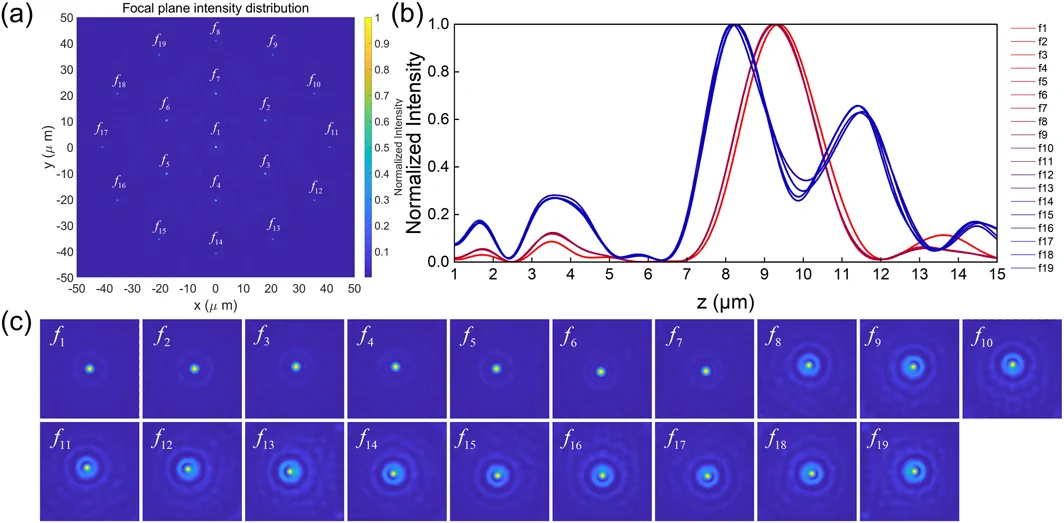
Simulated results of the fiber facet GO microlens array: (a) focal‐plane intensity distribution of the 19‐lens array (1 + 6 + 12, $f_{1}‐f_{19}$); (b) axial intensity distribution of each lens; (c) local intensity profiles (10 μm × 10 μm) of individual focal spots.

Figure 3a presents the intensity distribution in the *x*‐*y* focal plane extracted at the peak focal position of the central GO lens. The lenses are arranged from $f_{1}$ to $f_{19}$, where the focal intensity of the central lens is significantly higher than that of the peripheral lenses.

Figure 3b shows the calculated the normalized intensity on optical ax for each GO lens. Lenses $f_{1}-f_{7}$ correspond to the central GO lens and the six lenses located on the inner hexagonal ring. Their axial intensity profiles exhibit sharp and well‐aligned peaks, with the corresponding focal lengths tightly clustered around the designed value of 9 μm, showing slight fluctuations. This indicates a high degree of uniformity in the inner region, enabling precise and coplanar focusing. In contrast, lenses $f_{8}-f_{19}$, which are located on the outer dodecagonal ring, show a reduced alignment of the axial intensity peaks, accompanied by noticeable axial shifts of the focal positions. This focal length dispersion originates from the non‐ideal wavefront generated by the beam‐expanding fiber, where a larger phase gradient is present toward the outer region.

Figure 3c further provides a focal neighborhood analysis by calculating the intensity distribution within an area of 10 μm × 10 μm of each focus. The focal spots of $f_{1}-f_{7}$ are uniform, with low and symmetric sidelobe levels. By comparison, the focal spots of $f_{8}-f_{19}$ exhibit pronounced aberrations, characterized by spot broadening, reduced peak intensity, and asymmetric sidelobes. This behavior arises from the higher phase gradient of the output field from the beam‐expanding fiber at the periphery, which introduces initial wavefront distortions in the incident field received by the outer lenses. These distortions are subsequently amplified during the focusing process, leading to significant wavefront errors and a degradation of the focal quality.

## 3 Fabrication and Characterization

A 450 nm‐thick GO film was fabricated by vacuum filtration method and then transferred onto the fiber facet using the liquid transfer method. Detailed properties of the GO are shown in Supporting Information S1: Figures S3 and S4 and the properties of rGO are based on the ref. [43]. The thickness scans are shown in Supporting Information S1: Figure S4. The microlenses were directly patterned through femtosecond laser direct writing (FLDW, Innofocus Nanoprint3D) at 1030nm wavelength, 300fs pulse width and 25kHz repetition rate through a 100× objective (NA = 0.8). The optimized parameters were a scanning speed of 15 μm/s and an average power of 0.18mW.

Figure 4 presents the fabrication results and corresponding characterizations of the fiber‐integrated GO microlens array.

**FIGURE 4:**
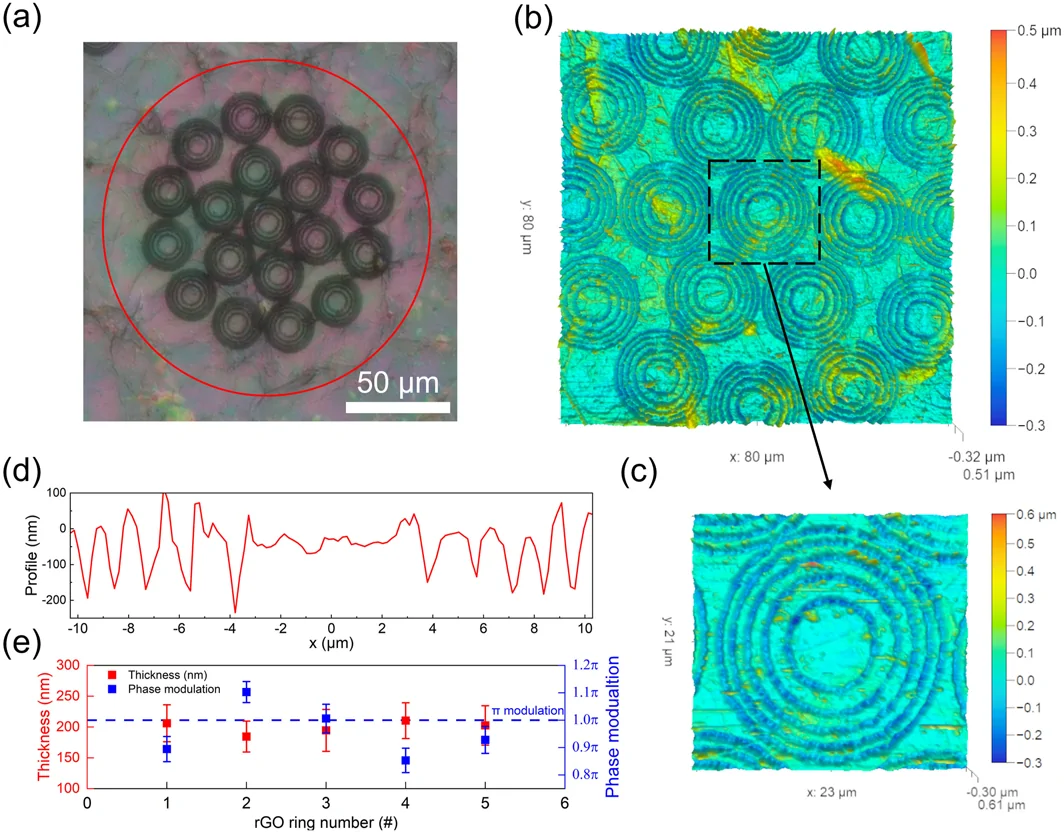
Characterization results of the fabricated fiber GO microlens array: (a) optical microscopy image of the 19‐lens array on the fiber facet; (b) large‐area 3D surface profile measured by AFM; (c) magnified 3D surface profile of central GO microlens; (d) cross‐sectional height profile across the GO lens (red line); (e) measured thickness and corresponding phase modulation for each rGO ring.

Figure 4a shows an optical microscopy image of the GO microlens array fabricated on the fiber facet. The red circle marks the coreless fiber region, whereas the outer area corresponds to the ceramic ferrule. A total of 19 five‐ring GO microlenses are patterned on the fiber facet. The microlenses are arranged according to the designed packing geometry, exhibiting accurate positioning and uniform spacing. The concentric ring structures of each GO microlens are clearly resolved, demonstrating the stability and repeatability of the femtosecond laser direct writing process, as well as good adhesion between the GO film and the fiber end face. No large‐area delamination or collapse of the GO film is observed.

Figure 4b displays the three‐dimensional surface height distribution of the GO microlens array measured by atomic force microscopy (AFM). Within the entire scanned area (80 μm × 80 μm), the relief structures corresponding to the 19 GO microlenses are clearly identified. The GO film is locally photoreduced to rGO by laser irradiation, resulting in a height difference of approximately 230 nm between the rGO and GO regions. The height distribution is relatively uniform across the array, indicating a good global consistency of the laser‐induced modification. The black dashed box highlights a magnified view of the central GO microlens.

Figure 4c shows the enlarged three‐dimensional surface morphology of the central GO microlens. Smooth transitions between adjacent rings are observed, confirming accurate radial modulation. Slight surface roughness is present, which can be attributed to the intrinsic inhomogeneity of the vacuum‐filtered GO film and localized laser–material interactions; however, this does not compromise the overall lens profile.

Figure 4d presents a one‐dimensional cross‐sectional height profile extracted along the red line in the *x*‐direction of the GO microlens shown in Figure 4c. The periodic height modulation corresponding to the concentric ring design is clearly evident. Although small fluctuations on the order of several tens of nanometers are observed, the overall modulation depth is well controlled. These fluctuations likely arise from nanoscale wrinkles in the GO film and energy variations during the scanning process. Their amplitudes are significantly smaller than the designed structural depth, and therefore have a limited impact on phase modulation.

Figure 4e summarizes the analysis of the GO microlens structure and its phase modulation performance. The red squares represent the central thickness of each rGO ring measured by a profilometer, whereas the blue squares indicate the corresponding phase modulation difference between the rGO rings and the surrounding GO film. The thickness of each rGO ring is designed to be around 200 nm, with only small deviations among different ring numbers, demonstrating good fabrication repeatability. Importantly, the extracted phase modulation for all rings is close to the target value of π, as indicated by the dashed reference line. This confirms that rGO provides sufficient phase modulation depth to achieve the intended constructive interference.

## 4 Results and Discussion

The focusing capabilities of the fiber GO lens array were characterized by a homemade 4‐f imaging system. The fiber OAM GO metalens was mounted on a custom‐made holder, and the far‐field intensity distribution was captured using a 100× objective lens with a NA of 0.8 and CCD camera operating at the specific wavelength. The system was designed to capture the image of the diffractive plane. To obtain intensity distributions at various cross‐sections along the *z*‐axis, a high‐precision motorized stage was used to scan along the optical axis. This scanning process enabled the reconstruction of complete far‐field intensity patterns by recording the focal field at different positions along the optical axis. The entire system was computer‐controlled.

Two types of laser sources were employed for the characterization: a distributed Bragg reflector (DBR) laser operating at 850 nm and distributed feedback (DFB) lasers operating at 1310 and 1550 nm.

The Figure 5 presents the focusing results of the fiber GO lens array under an 850 nm DBR laser. Figure 5a shows the normalized far‐field intensity distribution map, which is obtained through axial scanning through a 4‐f imaging system. The 19 designed focal points exhibit good consistency and uniformity, indicating the array possesses excellent phase modulation consistency and process repeatability. Figure 5b displays the intensity distribution results of each focal point from $f_{1}$ to $f_{19}$ on their respective focal planes, where the red curves represent the theatrical results and the blue curves denote the experimental results. Each subgraph corresponds to the normalized spatial intensity distribution of focal points $f_{1}$ to $f_{19}$ in the array at their axially optimal focal planes. As observed in Figure 5b, all focal points exhibit concentrated peak characteristics with good consistency in the FWHM, without obvious sidelobe overflow or intensity distortion.

**FIGURE 5:**
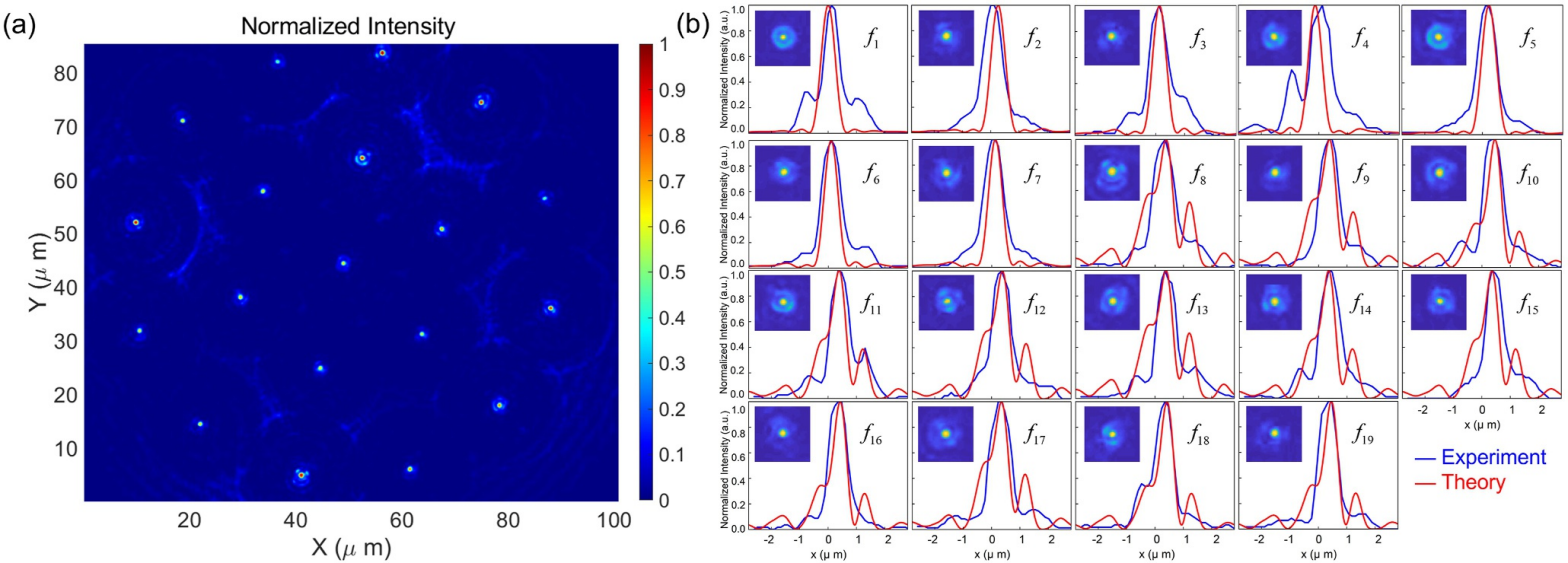
Focus characterization results of GO lenses array: (a) normalized far‐field intensity distribution at the focal plane; (b) comparison between experiment and theory focal profiles.

Among them, the focal points from $f_{1}$ to $f_{7}$ achieve ideal performance with excellent phase modulation accuracy and focusing effect; the outer focal points from $f_{8}$ to $f_{19}$ show significant distortion, with their intensity distribution deviating from the ideal concentrated mode and slight energy dispersion. This phenomenon may be attributed to the higher phase gradient at the edge of the beam‐expanding fiber compared to the center, leading to the failure of the GO lenses at the edge to achieve the required phase modulation. The experimental intensity distribution characteristics of all focal points align well with the theoretical predictions.

Figure 6 presents the experimental and theoretical comparison of focal lengths and NA for 19 focal spots of the fiber GO lens array, with the horizontal axis representing lens numbers and the vertical axis denoting focal lengths. Similarly, red dots indicate calculated focal lengths, whereas blue dots represent experimental results.

**FIGURE 6:**
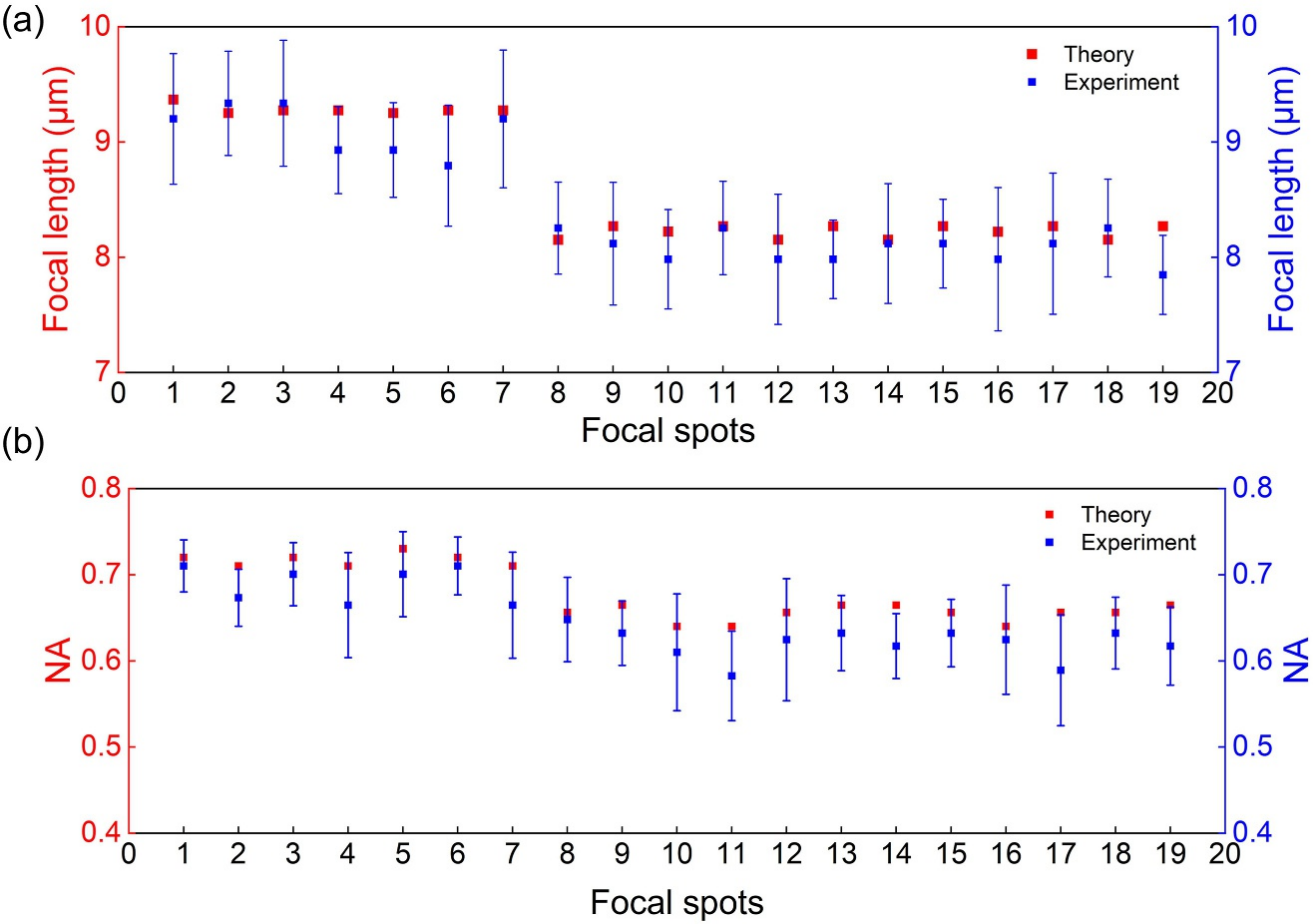
Experimental and theoretical comparison of focal lengths (a) and the NA (b) for 19 focal spots of the fiber GO lens array.

From the comparison, the experimental focal lengths of inner focal spots (from $f_{1}$ to $f_{7}$) show excellent agreement with theoretical calculations and are highly consistent with the designed target values. This demonstrates that the actual optical performance of lenses in this region is well‐aligned with the theoretical design, further verifying the precision of phase modulation and the reliability of process fabrication for the GO lenses, which strongly correlates with the superior intensity distribution of inner focal spots reported earlier. For outer focal spots (from $f_{8}$ to $f_{19}$), although the experimental and theoretical focal lengths still maintain a certain degree of matching, the deviation between the designed value and the results still exists. This phenomenon is consistent with the cause of intensity distortion in the outer focal spots—the great phase gradients of the beam‐expanding fiber at the edge not only disrupt the ideal focusing state of the optical field energy but also lead to deviations of the actual focal lengths from the designed theoretical values. This result also confirms the rationality of the theoretical prediction regarding focal length deviations in outer focal spots induced by phase distortion.

In addition to the focal intensity profiles, we quantitatively evaluated the power distribution and transmission efficiency of the 19 lens array. The total insertion loss at 850 nm is 1.85 dB. For each focal spot, we calculated the integrated intensity within the FWHM circular region. The ideal uniform splitting ratio for 19 beams is 1/19, centering at the peak, and normalizing by the total integrated intensity over the entire focal plane. Supporting Information S1: Figure S1 shows the extra loss of each focal spot. The central lenses exhibit an extra loss of 5 dB, indicating good power uniformity, whereas the outer lenses suffer from an extra loss up to 9 dB.

To quantitatively understand the degradation of the outer lenses, we characterized the phase profile of the expanded beam at the fiber facet. Supporting Information S1: Figure S1 presents the intensity and phase distributions of the output field. The radial phase gradient dϕ/dr, shown in Supporting Information S1: Figure S2b, increases from 0.1 rad/μm in the central region (30 μm) to a mean value of 0.2 rad/μm in the outer annulus. This steep phase gradient at the edge corresponds to a strong local wavefront tilt, which directly degrades the focusing performance of the outer lenses.

Notably, despite the focal length deviations in outer focal spots, the variation trend of all experimental data is consistent with the theoretical curve without irregular fluctuations, indicating that the deviations are caused by predictable structural or process factors rather than flaws in the design principle.

In Figure 6b, both the NA and focal length results show consistent trends. For inner focal spots $f_{1}$ to $f_{7}$, the experimental NA values, spanning 0.67–0.74, correspond to the tight focusing and match the theoretical predictions. For outer focal spots $f_{8}$ to $f_{19}$, the experimental NA values, spanning from 0.60 to 0.68, generally follow the theoretical trend but with slight deviations. These deviations are consistent with the influence of the large phase gradients at the fiber edge, which also affect the effective NA.

Alignment tolerance of the device is governed by the depth of focus (± 1.1 μm) and lateral spot size (± 0.9 μm) at NA = 0.70 and λ = 850 nm. The femtosecond‐laser‐reduced GO/rGO structure is robust, tolerating temperatures up to 180°C and mechanical bending [44]; the all‐fiber architecture resists vibrations, and a low‐refractive‐index polymer coating can be applied for long‐term environmental protection without altering the phase modulation. The mask‐free, digitally controlled femtosecond writing process requires only 90 seconds per lens, making it suitable for rapid prototyping, and it is readily scalable to higher throughput through parallel writing or replication techniques.

Figure 7 presents the focusing results of the fiber GO lens array under 1310 and 1550 nm DFB lasers. Figure 7a,c shows the normalized far‐field intensity distribution and focus of each spots on the focal plane obtained via axial scanning with a 4‐f imaging system at 1310 nm, respectively. Figure 7b,d are the corresponding distribution at 1550 nm.

**FIGURE 7:**
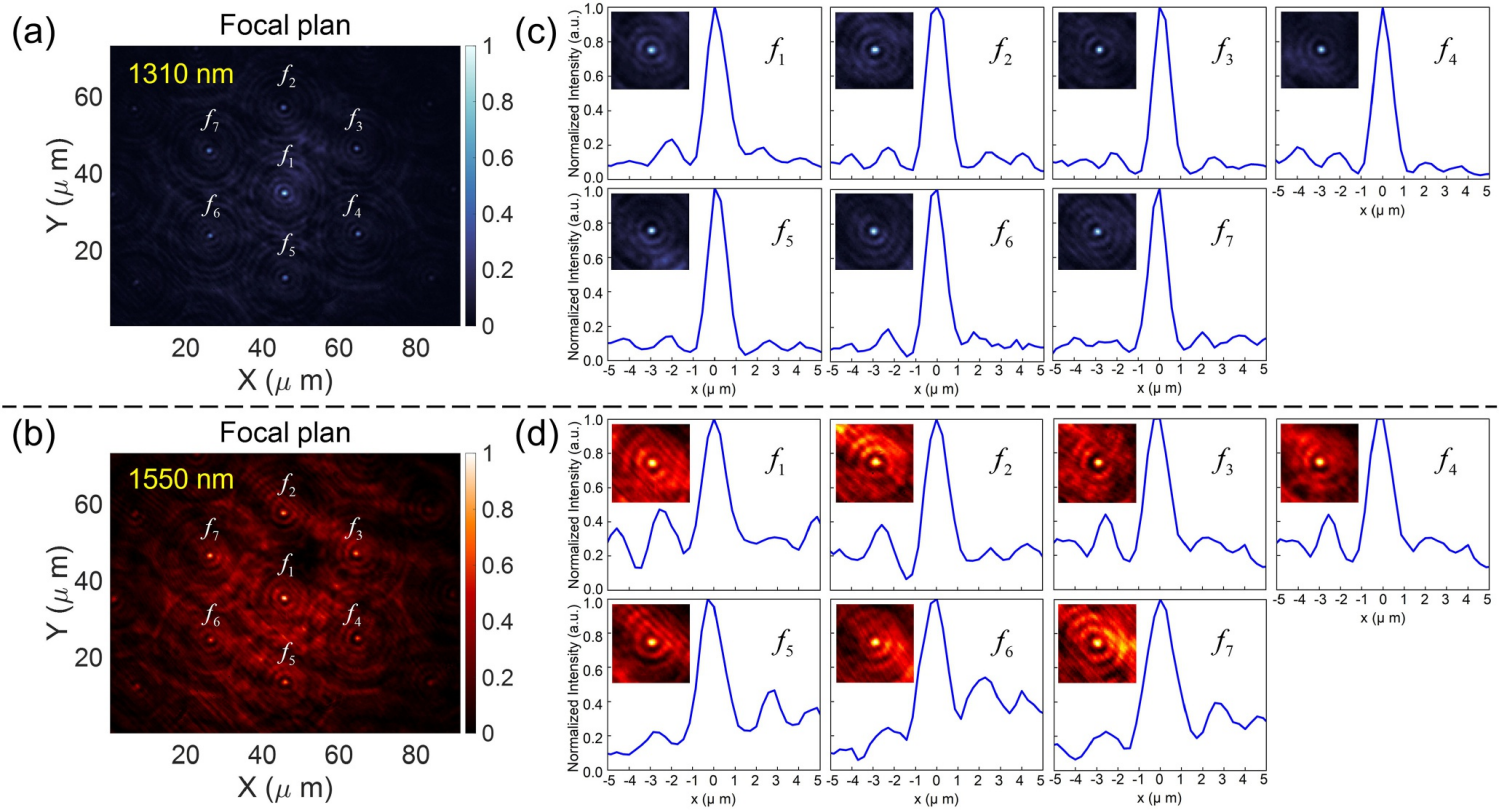
Focus characterization results of GO lenses array: (a) 1310 nm and (b) 1550 nm show the normalized far‐field intensity distribution at the focal plane; (c) 1310 nm and (d) 1550 nm show comparisons between experiment and theory focal profiles.

Due to significant edge distortion, notably lower intensity at the edges than the center, and the limitations of the infrared CCD (insufficient dynamic range and field of view), the test results of outer focal spots were not considered. As observed in Figure 7a,b, the focal spots formed by the array under both wavelengths exhibit spatial arrangement and good overall consistency, indicating the fiber GO lens array possesses stable phase modulation consistency and process compatibility in the 1310–1550 nm band, with no significant degradation of optical performance due to wavelength variation. Figure 7c,d display the normalized intensity spatial distributions of focal spots from $f_{1}$ to $f_{7}$ at their axially optimal focal planes for 1310 and 1550 nm, with the curves clearly presenting the experimental results. It can be seen that all tested focal spots under both wavelengths show distinct concentrated peak characteristics, regular FWHM, and no obvious sidelobe overflow or energy dispersion. This confirms the array exhibits excellent focusing performance and precise phase modulation capability in both the 1310 and 1550 nm bands, expanding its application potential in multi‐band optoelectronic systems. Table 1 compared with previous works (*means this is the efficiency of an individual lens. The efficiency range for the 19 lenses is provided), our device simultaneously achieves high NA, large array number, broadband operation, and single‐step fabrication on a fiber facet, representing a significant advance in compact integrated photonics.

**TABLE 1:** Comparison of GO lens.

	Function	Thickness	Substrate	NA	Efficiency	Elements	Diameter	Working wavelength
Zheng et al. [43]	Single lens	200 nm	Glass	0.8	32%	1	8 μm	400–1500 nm
Lee et al. [39]	Lens array	150 nm	Polymer	0.3	N.A.	5×5	800 μm	638 nm
Low et al. [40]	Single lens	900 nm	Glass	N.A.	4.5%	1	650 μm	638 nm
Chen et al. [41]	Single lens	400 nm	Fiber	0.89	7.2%	1	20 μm	850 nm
Chen et al. [45]	Single lens	400 nm	BE fiber	0.55	34.7%	1	100 μm	850 nm
Chen et al. [42]	OAM lens	400 nm	BE fiber	0.85–0.93	N.A.	1	100 μm	850–1625 nm
This work	Lens array	400 nm	BE fiber	0.67–0.74	9.8%–35.4%*	19	100 μm	850–1550 nm

## 5 Conclusion

In this work, we have successfully developed an ultra‐compact fiber facet GO microlens array using FLDW technology, realized miniaturization, integration density, and fabrication simplicity on fiber facet. The device achieves simultaneous beam splitting and focusing on a single fiber facet. The ultrathin GO film ensures minimal device thickness and efficient phase modulation through laser‐induced reduction to rGO. The results demonstrates broadband compatibility (850 nm, 1310 nm, and 1550 nm) with stable multi‐focus output. The GO‐based platform offers advantages such as single‐step fabrication, ultrathin structure, and high design flexibility. This work paves the way for the development of fiber‐integrated devices for various applications in integrated photonics and nanophotonics.

## Author Contributions

**Xiaoke Chen:** conceptualization, investigation, writing – original draft, writing – review and editing, visualization, methodology, formal analysis, data curation, software. **Lin Ma:** resources, funding acquisition, writing – review and editing, validation, project administration, formal analysis, supervision, visualization. **Linbin Bai:** resources, supervision, formal analysis, project administration, validation. **Tianqi Cheng:** writing – original draft, writing – review and editing, methodology, validation. **Zuyuan He:** resources, supervision, project administration, formal analysis, writing – review and editing.

## Supporting information


Supporting Information S1

## Data Availability

The data that support the findings of this study are available from the corresponding author upon reasonable request.
