# Supplementary material for: Ultra Compact and Broadband Fiber Facet Graphene Oxide Microlens Array Fabricated on a Beam‐Expanding Fiber Facet Using Femtosecond Laser Direct Writing Technique
Source: Nanophotonics. 2026 Jul 29;15(15):e70217. doi: 10.1002/nap2.70217 (PMC13642100; doi:10.1002/nap2.70217)
Supplement: Supplementary file 1 [file NAP2-15-e70217-s001.docx]

# Supplementary Material

Figure S1. Theory and experiment extra loss of 19 focal spots

Figure S2. Wavefront at the fiber facet (a) Wrapped phase distribution. (b) Radial phase gradient. (c) Normalized intensity. (d) Radial intensity and phase profiles.

Figure S3. Refractive and extinction coefficient of the GO film of our measured data.

Figure S4. The relationship between the thickness of the GO film versus phase modulation and the absorption.
